# Metastatic gallbladder cancer to the ovary presenting as primary ovarian cancer: a case report

**DOI:** 10.1186/s13256-021-03001-2

**Published:** 2021-08-05

**Authors:** Chorong Kim, Yoon Hyeon Hu, Kyoungyul Lee, Hyang Ah Lee, Dong Hun Lee, Yung-Taek Ouh

**Affiliations:** 1 Department of Obstetrics and Gynecology, School of Medicine, Kangwon National University, 156, Baengnyeong-ro, Chuncheon-si, Kangwon Republic of Korea; 2 Department of Pathology, Graduate School of Medicine, Kangwon National University, Chuncheon-si, Kangwon Republic of Korea

**Keywords:** Gallbladder neoplasms, Krukenberg tumor, Neoplasm metastasis

## Abstract

**Background:**

Krukenberg tumors are uncommon and are indicative of an ovarian metastatic carcinoma that originates from another site of primary malignancy. The majority of metastases to ovaries are derived from the stomach and colon. We present a rare case of a metastatic ovarian malignant tumor that originated from gallbladder adenocarcinoma.

**Case presentation:**

A 45-year-old premenopausal Korean woman presented with abdominal distension. Bilateral multiseptated ovarian tumors and a wall-thickened gallbladder were found on abdominal computed tomography. The patient was diagnosed with metastatic ovarian carcinoma arising from gallbladder adenocarcinoma and was treated with adjuvant chemotherapy.

**Conclusions:**

Metastases to the ovaries from other sites, including the gallbladder, are rare and usually resemble primary ovarian tumors. Therefore, potential metastatic ovarian tumors of newly diagnosed pelvic masses should be considered in differential diagnoses.

## Background

The ovary is a common site of metastasis from primary tumors in the stomach, large bowel, appendix, breast, and uterus [1]. An adnexal mass that is suspected as an ovarian neoplasm will be determined to be metastatic in approximately 10–25% of all ovarian malignancies [1]. The Krukenberg tumor is a classic metastatic lesion to the ovary that is derived from a primary malignancy. The majority of Krukenberg tumors originate from the stomach, followed by the colon [2]. Gallbladder and extrahepatic bile duct malignancies are rarely implicated in ovarian tumors [3]; however, they significantly worsen the prognosis. We present a rare case of a Krukenberg tumor arising from a gallbladder carcinoma.

## Case presentation

A 45-year-old multiparous Korean woman with regular menstrual cycles presented with dyspepsia and abdominal distension. On physical examination, the abdomen was distended with a tense cystic mass arising from the pelvis, corresponding to a 20-week gravid uterus, which was palpable bimanually. Liver function tests showed normal ranges, including total bilirubin (0.4 mg/dL), aspartate aminotransferase (27 U/L), alanine aminotransferase (27 U/L), and alkaline phosphatase (52 U/L). The serum level of cancer antigen 125 (CA125) was increased to a level of 61.6 U/mL, and cancer antigen 19-9 (CA 19-9) was in the normal range (8.6 U/mL).

**Fig. 1:**
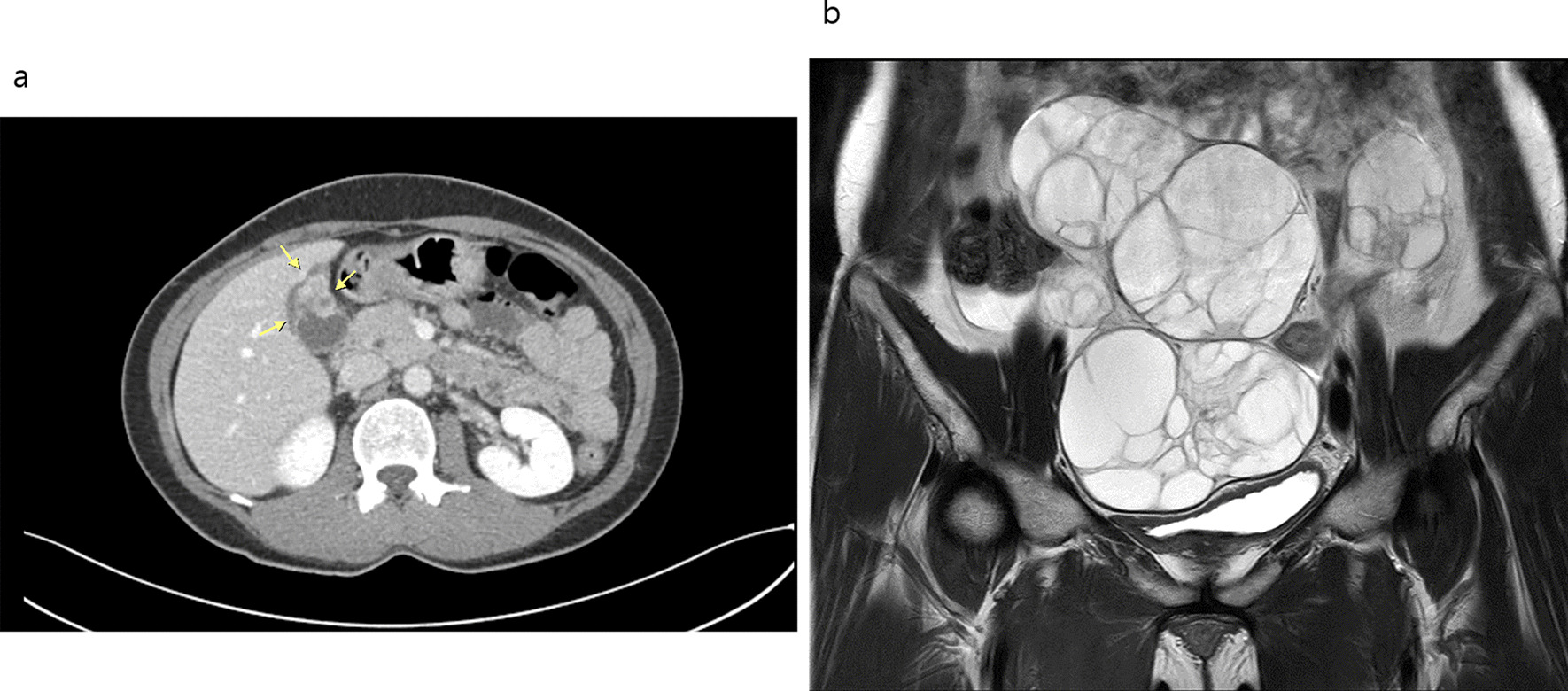
Magnetic resonance imaging findings of large bilateral ovarian tumors (**a**) and abdomen and pelvis computed tomography scan with intravenous contrast demonstrating wall thickening of the gallbladder (**b**) (arrows indicates wall-thickened gallbladder)

Large multiloculated ovarian masses, measuring 16 × 15 × 11 cm on the right side and 5 × 5 × 4 cm on the left side were detected on magnetic resonance imaging (Fig. 1a). A subsequent computerized tomography (CT) scan of the abdomen showed diffuse wall thickening of the gallbladder fundus and body portion without distension (Fig. 1b). The imaging and test results suggested primary ovarian cancer with an incidental focal adenomyomatosis. Additional lesions were not detected by gastrointestinal fiberscopy and colonoscopy.

A laparotomy was performed based on the preoperative diagnosis of a primary ovarian malignant neoplasm with pathologic finding of gallbladder. The patient underwent a total abdominal hysterectomy, bilateral salpingo-oophorectomy, bilateral pelvic lymphadenectomy, paraaortic lymphadenectomy, omentectomy, and peritoneal washings for cytology. A large multilobulated well-capsulated neoplasm of both ovaries was found, and mucinous adenocarcinoma was detected in both ovaries through a frozen biopsy. No peritoneal dissemination was observed. Immediately following the gynecological operation, the patient underwent radical cholecystectomy by hepatobiliary pancreas surgery team.

**Fig. 2:**
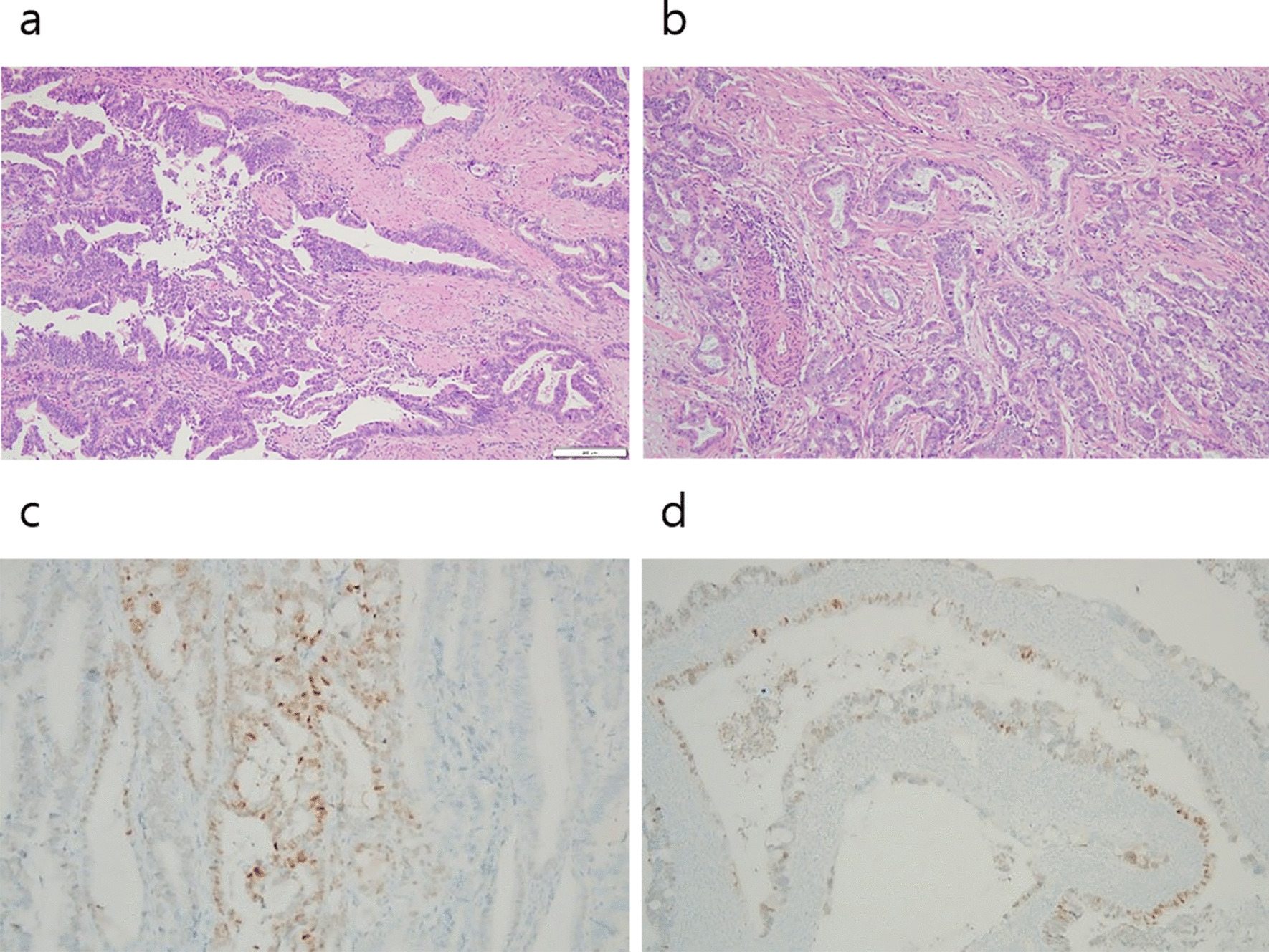
Histopathologic finding of gallbladder and ovarian tumors. **a** Adenocarcinoma of gallbladder (hematoxylin and eosin stain, ×100), **b** mucinous adenocarcinoma of ovary (hematoxylin and eosin stain, ×100), **c** some tumor cells in gallbladder are positive for CDX2 immunohistochemical stain (×200), **d** some tumor cells in ovary are positive for CDX2 immunohistochemical stain (×100)

Adenocarcinoma was detected in the gallbladder (Fig. 2a). Microscopic examination of both ovarian neoplasms showed bilateral mucinous adenocarcinoma (Fig. 2b), which was a metastasis from the gallbladder. Immunohistochemistry showed that gallbladder adenocarcinoma was focally positive for CDX2 (Fig. 2c). Ovarian adenocarcinoma was also positive for CDX2 (Fig. 2d) while negative for an estrogen receptor. Lymph node metastasis was noted in one left external iliac lymph node.

Following surgery, the patient had adjuvant chemotherapy with six cycles of cisplatin and gemcitabine. There was no clinical and radiologic recurrence noted at the 12-month follow-up after surgery.

## Discussion

Differential diagnoses of primary and metastatic tumors often present diagnostic difficulties for both clinicians and pathologists. Metastatic tumors can be confused with primary tumors; however, it is important to distinguish between them because the treatment approaches can differ. In addition to low abdominal pain, patients frequently report symptoms of postmenopausal bleeding, weight loss, nausea, vomiting, and abdominal distension [4]. Primary malignant tumors from the colon, appendix, and upper gastrointestinal tract are the most common primary malignancies that are associated with clinical findings of primary ovarian cancer [5]. A previous study found that gallbladder primaries made up 1.7% of all metastatic lesions to the ovary [4].

The pathway for ovarian metastases from the gastrointestinal tract is still unknown. In previous studies of metastatic ovarian cancer that originated from colon cancer, most cases were determined to be metastases to the ovarian stroma [6]. Laterality of the ovary involved with the metastasis was not associated with the site of the primary tumor. In addition, lymph node metastasis was frequently found in Krukenberg tumors [7]. This suggests that metastasis was through retrograde lymphatic flow, indicating that the ovary was frequently involved as a retroperitoneal organ.

In general, metastatic ovarian cancers are smaller in size than primary ovarian cancers and usually contain cysts [8]. The tumors are typically smaller than 10 cm in diameter. Ovarian malignant tumors that occur bilaterally are common in metastatic ovarian cancer, and they have been reported to occur bilaterally in more than 80% of all Krukenberg tumors [9]. Therefore, tumor size and bilaterality provide clues for distinguishing metastatic ovarian cancer from primary ovarian cancer [10]. The gross morphology that favors metastases includes a relatively small size (< 10 cm), involved bilateral ovaries, a nodular growth pattern, and tumors on the surface or cortex of the ovary [10].

Because bilateral ovarian mucinous adenocarcinoma is very rare, the possibility of metastasis from other organs should be ruled out as part of the diagnosis. Immunohistochemical staining is helpful for the differential diagnosis of primary versus metastatic tumors. Cytokeratins (CK) 7 and 20 are commonly used markers in ovarian tumors [9]. Primary ovarian cancers are almost always positive for CK7 but are typically negative for CK20. It is also known that more than 80% of serous and endometrioid ovarian carcinomas are positive for estrogen receptors (ERs) [11]. CDX2 is usually negative for primary ovarian cancer, whereas CDX2 is positive for ovarian metastases from the colon cancer or gallbladder [12]. In our case study, the bilateral ovarian mucinous adenocarcinoma had an identical immunoprofile with gallbladder cancer.

There is currently no consensus on the treatment approach for metastatic ovarian cancer, especially in metastases from the gallbladder. The first step is to identify the primary tumor site, which should be managed according to histologic type and stage [1]. To improve the survival rate, the gold standard treatment for patients with Krukenberg tumors in the absence of dissemination of the carcinoma or pleural effusion is optimal cytoreductive surgery. Adjuvant or palliative chemotherapy can also be administered.

## Conclusion

Metastases to the ovary often pose diagnostic problems for both clinicians and pathologists. While some metastatic lesions are clearly secondary findings in patients with disseminated disease, others may be confused with primary tumors, and it is important to identify the therapeutic considerations for the latter group. Consideration of metastatic ovarian cancer in addition to primary ovarian cancer are important in the differential diagnosis of ovarian tumors, and collaboration between radiologic and pathologic evaluations is essential to determine the best treatment options for the patient.

## Data Availability

The dataset(s) supporting the conclusions of this article is (are) included within the article.
